# Early recognition of hypohidrotic ectodermal dysplasia

**DOI:** 10.1186/1746-160X-8-S1-I2

**Published:** 2012-05-25

**Authors:** Holm Schneider

**Affiliations:** 1 Department of Pediatrics, University Hospital Erlangen, Germany

## 

Hypohidrotic ectodermal dysplasia (HED) is characterized by severe hypohidrosis, hypoplasia of sweat, sebaceous, submucous, meibomian and mammary glands, hypotrichosis, and oligodontia. In early childhood, HED is a life-threatening disorder based on the risks for hyperthermia and pneumonia. Awareness of the hazards related to this rare genetic disease is most helpful in preventing avoidable calamities. Prognosis therefore depends on the time point of diagnosis.

Typical facial features (frontal bossing, sparse eyebrows and eyelashes, wrinkling and hyperpigmentation of the periorbital skin, saddle nose, everted lips, hypoplastic mandible) may allow immediate postnatal recognition of HED, if the disease is known to the family or the doctor. Molecular analysis of one or more of the 4 candidate genes *EDA*, *EDAR*, *EDARADD* and *NEMO* can then be arranged to make a definitive diagnosis. Infants with HED may also be identified by their inability to sweat. A survey conducted among parents of 100 children with ectodermal dysplasia registered with the German-Swiss-Austrian patient support group revealed that almost all parents had observed episodes of unexplained fever during the first year of life. If infants had to be placed in an incubator after birth, body temperature recording proved to be of utmost importance and often allowed early clinical diagnosis of HED. Furthermore, recent studies of our group confirmed a consistent, quantifiable defect of sweat gland function in male individuals with HED as a disease biomarker. Pilocarpine-induced sweat volume, palmar or plantar sweat duct density and skin conductance before and after stimulation were determined also in newborn infants. Genotype-phenotype correlation was seen across all measurements of sweat gland function, but, surprisingly, not with respect to the number of sweat glands (which can be determined very soon after birth) and the degree of oligodontia (diagnosable already by prenatal ultrasonography). This is important, since such easily recognizable signs obviously do not allow prediction of the risk of hyperthermia and the associated morbidity and mortality in HED patients.

